# How Watching Pinocchio Movies Changes Our Subjective Experience of Extrapersonal Space

**DOI:** 10.1371/journal.pone.0120306

**Published:** 2015-03-23

**Authors:** Chiara Fini, Giorgia Committeri, Barbara C. N. Müller, Eliane Deschrijver, Marcel Brass

**Affiliations:** 1 Department of Experimental Psychology, Ghent University, Henri-Dunantlaan 2, 9000, Ghent, Belgium; 2 Laboratory of Neuropsychology and Cognitive Neuroscience, Department of Neuroscience and Imaging, University G. d’Annunzio, Chieti-Pescara, Italy; 3 Institute for Advanced Biomedical Technologies, Foundation University G. d’Annunzio, Chieti, Italy; 4 Behavioural Science Institute, Radboud University Nijmegen, PO Box 9104, 6500 HE Nijmegen, The Netherlands; University G. d'Annunzio, ITALY

## Abstract

The way we experience the space around us is highly subjective. It has been shown that motion potentialities that are intrinsic to our body influence our space categorization. Furthermore, we have recently demonstrated that in the extrapersonal space, our categorization also depends on the movement potential of other agents. When we have to categorize the space as “Near” or “Far” between a reference and a target, the space categorized as “Near” is wider if the reference corresponds to a biological agent that has the potential to walk, instead of a biological and non-biological agent that cannot walk. But what exactly drives this “Near space extension”? In the present paper, we tested whether abstract beliefs about the biological nature of an agent determine how we categorize the space between the agent and an object. Participants were asked to first read a Pinocchio story and watch a correspondent video in which Pinocchio acts like a real human, in order to become more transported into the initial story. Then they had to categorize the location ("Near" or "Far") of a target object located at progressively increasing or decreasing distances from a non-biological agent (i.e., a wooden dummy) and from a biological agent (i.e., a human-like avatar). The results indicate that being transported into the Pinocchio story, induces an equal “Near” space threshold with both the avatar and the wooden dummy as reference frames.

## Introduction

We perceive space as a function of our action potentialities [1–3]. According to the most recent version of the embodied perception theory visual information is *scaled by* the non-visual metrics derived from the body [4]. Because each individual has different physical features, like for example a different length of the limbs, perception of space is to some degree idiosyncratic, and can be considered a phenotypic expression [4].

Some studies on distance perception in the extrapersonal space have shown that when the observer has to carry a heavy load, the egocentric distance to a given object is perceived to be larger [1]. In the same way, the motoric decline associated with aging leads old people to consider a distance as farther compared to younger individuals [5]. Interestingly, we also take into account bodily resources of others when we are asked to categorize the space between another individual as an allocentric reference frame (RF) and an object. Indeed, the extrapersonal space categorized as “Near” is wider when the other agent has the appearance of a biological agent that is able and prone to walk towards a target in the extrapersonal space. When we adopt a non-biological body (i.e., a wooden dummy) as RF instead, the space categorized as “Near” is smaller [6]. Hence, recognizing the biological nature of the agent seems to be essential in order to categorize a wider portion of space as “Near”. This interpretation is in accordance with research on action co-representation, which reveals that we represent biological agents in a different way than non-biological agents [7–11].

In the study described above, however, the appearance of the wooden dummy differed from the appearance of the human body. Therefore it is unclear whether the effect depends on perceptual characteristics of the agent or on the abstract attribution of action potentialities and intentions to the RF.

It is noteworthy that the perception of the agent's action potentialities and intentions can be manipulated without confounding the appearance of the agent itself. It has been demonstrated, for example, that the degree to which action observation influences action execution depends on whether or not participants believed that a moving hand wearing a leather glove belongs to a biological agent or to a non-biological agent [12, 13]. Moreover, Müller and colleagues (2011) have shown that it is possible, through a top-down belief manipulation, to influence the action co-representation of a non-biological interaction partner. Participants had to form a vivid impression of their interaction partner by watching a short video fragment with either a human or an animated character—Pinocchio—as its main character. Afterwards, they performed a go/no-go Simon task, interacting with either a human or a wooden dummy. In the case of the Pinocchio manipulation, the non-biological agent was co-represented in the social Simon task, with a consequent suppression of the co-representation of the biological agent.

The aim of the current experiment was to manipulate the belief about the action potentialities and intentions of the non-biological RF (i.e., the wooden dummy) while participants carry out an extrapersonal space categorization. Transportation or absorption into a narrative story can strongly influence perception of the world, even changing people's beliefs about what is real [14, 15]. Therefore, participants in this study were asked to “transport” themselves in the Pinocchio story. To do so, they read and watched a fragment of the Pinocchio story. We expected that the degree of transportation, as measured by a questionnaire [14], would predict a reduction of the difference between the “Near” space threshold when adopting the non biological agent (i.e., a wooden dummy) compared with a biological agent (i.e., an avatar) as allocentric RF. More specifically, participants with the highest transportation score might show an equal “Near” space threshold with the non-biological agent as the RF, compared with the biological agent. Conversely, participants with the lowest transportation score would show a larger “Near” space threshold when adopting as RF the biological agent, as compared to when the non-biological agent was adopted as the RF.

## Materials and Methods

### Participants

Sixty-six students from Ghent University (49 females, all but 5 right-handed; mean age 21,6 years; range 18–30) with normal or corrected-to-normal vision participated in the study, for which they received financial compensation.

### Ethics Statement

Participants provided written informed consent before the beginning of the experiment, which was approved by the local Ethics Committee, and was conducted in accordance with the ethical standards of the 1964 Declaration of Helsinki.

### Procedure

**Fig 1:**
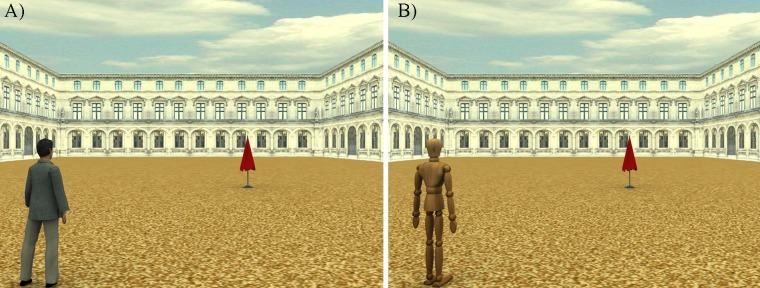
Stimuli in 3D scenario used in the experiment. A) Biological agent RF. B) Non-biological agent RF.

Participants were asked to read a short summary about a 5-minute video fragment of Walt Disney's Pinocchio. Afterwards, they watched the corresponding video fragment [16],and filled in a questionnaire which measured the degree of the participant's transportation in the Pinocchio story—specifically by exploring the attributed agency and intentionality to the characters of Pinocchio (Q1, Q2, Q4, Q5) and Geppetto (Q3), and by exploring the degree of identification with the same characters (Q6, Q7) (Green & Brook 2000) using a 7-point Likert scale (1 = do not agree, 7 = agree) (Table 1). Subsequently, the participants were asked to perform an extrapersonal space categorization task. Stimuli included 3D scenes created by means of a 3D modelling software (3D studio Max 4.2, Autodesk, Discreet). The scene was a 3D environment, representing a square arena suitable to be travelled by feet, defined by the two short lateral wings and the long central wing of a palace (Fig. 1). In the first set of stimuli (Fig. 1A) a biological agent (i.e., a human-like avatar) was located 45° to the right (left) of the central camera representing the participant’s perspective, and a red beach umbrella serving as a target was located along a central vector aligned with the avatar at 27 different distances (from 2 m to 54 m). The second set of stimuli (Fig. 1B) was identical to the first one, except for the presence of a non-biological agent, (i.e., a standard and featureless wooden dummy) instead of the avatar. The avatar and the dummy were equally tall (177 cm) and had the same anterior spatial extension. We administered the stimuli through the limit method. This is a method for measuring perceptive thresholds, in which the subject is typically presented with series of stimuli in stepwise increasing or decreasing intensity (distance in our case), until an intensity change is felt and consequently reported. In our experiment, a series of trials consisted of 27 potential images, each with a red beach umbrella at a continuously increasing distance from the reference frame. A series started with a white fixation cross (1,5° x 1,5° cm) on a black background (2500 ms). An experimental image lasted 2500 ms and was followed by a white fixation cross on a black background for 2500 ms, after which the next trial was presented.

**Table 1:** Questionnaire statements and mean scores for each item in Low Transportation (LT) and High Transportation (HT) groups.

**Questionnaire statements**	**LT<25 percentile**	**HT>75 percentile**
Q1: “During the vision of the movie I could construct a representation of Pinocchio as living one”	4,45 (1,03)	5,63 (0,92)
Q2: “During the vision of the movie I had the impression that Pinocchio had its own will”	2,72 (1,10)	6,36 (0,92)
Q3: “During the vision of the movie I could construct a representation of Geppetto as living one”	5,18 (0,87)	6,36 (0,92)
Q4: “During the vision of the movie I had the impression that Pinocchio followed its own intentions”	3,09 (1,13)	6,45 (0,68)
Q5:”In the movie Pinocchio behaved as a normal person”	2,73 (1,00)	5,63 (1,20)
Q6:”I'm identified in Pinocchio”	2,00 (0,89)	5,00 (1,34)
Q7:”I'm identified in Geppetto”	3,45 (1,43)	4,36 (2,11)

Subjects were asked to categorize the red beach umbrella as “Near” or “Far” from the two different RFs, by pressing two different buttons arranged horizontally on the computer keyboard and counterbalanced in the “Near”/“Far” judgment. The “Near”/“Far” judgments were requested as immediate and subjective, and it had to be expressed during the presence of the image on the screen.

In ascending series, the red umbrella was progressively moved away from the RF until the participants provided three consecutive “Far” judgments. In descending series, the red umbrella was progressively moved closer to the RF until the participants provided three consecutive “Near” judgments. This was done to ensure judgments consistency.

Subsequently, the following series started. The point where participants expressed a transition from “Far” to “Near” (descending series) and from “Near” to “Far” (ascending series), was called *Judgment’s Transition Threshold* (JTT). The JTT was calculated for each subject. Series were averaged together to obtain a final mean JTT referring to the different RFs. Higher JTT values show a categorization of space as “Near” at longer target distance, as compared to lower JTT values. In other words, the higher the JTT, the broader is the space categorized as “Near”. Each series was repeated 4 times for each RF. Each subject was thus submitted to 16 randomized experimental series (2 RFs: Biological agent, Non-Biological agent x 8 series type: 4 ascending, 4 descending). Before starting the experiment, we instructed the subjects about the task to perform and presented them the two sets of stimuli once.

Stimuli were presented at full screen on a 15’ computer display placed 57 cm from the subject. The presentation of the stimuli and the recording of the participant’s responses were controlled by means of the program Presentation (version 14.9.08.11)

### Data analysis

In order to investigate whether transportation changes the space threshold for the non-biological agent relative to the space threshold for the biological agent we generated a difference score. In particular, we computed an index of distance judgment as the difference between the non-biological agent (NB) and the biological agent (B) space threshold (JTT^NB-B^). Positive values indicate a smaller “Near” space categorization adopting as RF a biological agent with respect to a non-biological agent, while negative values indicate a wider “Near” space categorization adopting as RF a biological agent compared to a non-biological agent.

We investigated the relationship between transportation and space categorization in two different ways. First, we correlated the distance judgment index (JTT^NB-B^) with the Transportation scores obtained in the questionnaire. Then, we selected the 25% of participants with the highest transportation scores (High Transportation group, HT) and the 25% participants with the lowest transportation scores (Low Transportation group, LT), and included them as a between-subject factor in a mixed ANOVA. The latter analysis was done in order to clarify the nature of the correlation between transportation and space categorization with the two RFs (e.g., space extension for the non-biological agent vs. space reduction for the biological agent).

## Results

Three subjects were excluded because they scored ± 2 SD from the mean questionnaire score.

**Fig 2:**
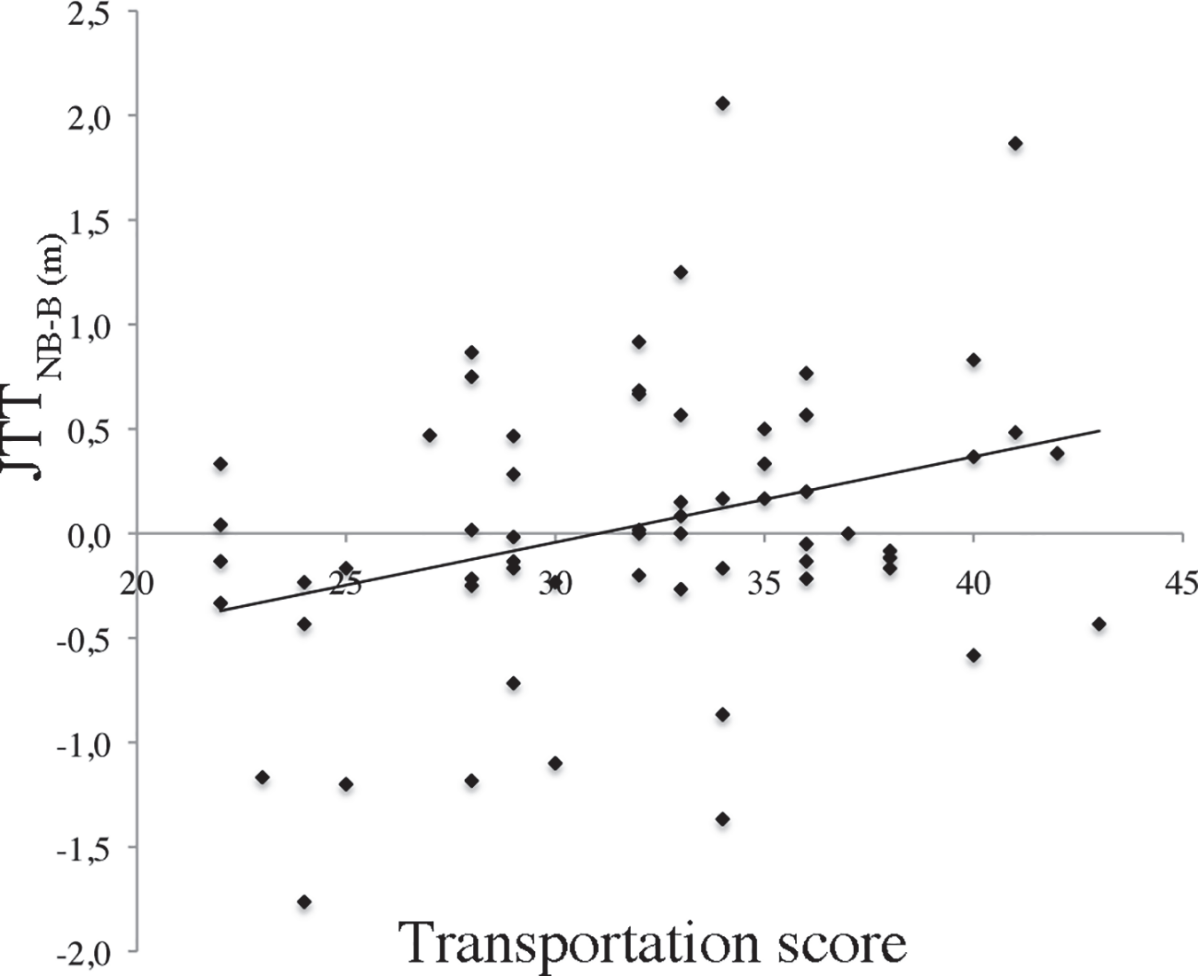
Significant correlation between the JTT^NB-B^ index and the questionnaire transportation score.

A significant Pearson’s correlation was found between the Transportation score and the JTT^NB-B^ index (r = .32 *p* = .009) (Fig. 2). The Transportation score positively predicted a reduction of “Near” space threshold when the biological agent was adopted with respect to the non-biological agent as RF. In other words, the higher degree of transportation in the Pinocchio story, the smaller the space categorized as “Near” when the biological agent was adopted as RF compared to the non-biological agent. To clarify the role played by the mechanism of identification with the characters, an additional correlation has been performed between the Transportation score without the last two items (i.e., “I’m identified in Pinocchio”; “I’m identified in Geppetto”) and the JTT^NB-B^ index (r = .27, *p* = .029). The significant correlation found without the identification items suggests that the mechanism behind the reduction of the “Near space extension” for the biological agent compared with the non-biological agent is mainly driven by the general transportation into the story.

In order to investigate the importance of perceiving Pinocchio as an intentional agent, three items concerning the agency (i.e. “During the vision of the film I have the impression that Pinocchio had his own will”, “During the vision of the film I have the impression that Pinocchio followed its own intentions”, “In the film Pinocchio behaved like a normal person”) have been entered into a correlation with the JTT^NB-B^ index. The marginally significant correlation (r = .24, *p* = .055) indicates that the agency attributed to Pinocchio plays a role in inducing the effect.

**Fig 3:**
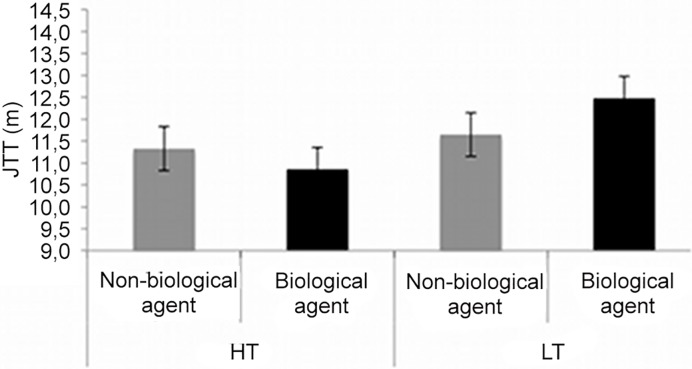
Mean judgment transition threshold (JTT) in LT and HT groups in the two RF types (Biological and Non-biological agent).

Moreover, a mixed ANOVA with the between-subject factor Group (HT, LT; see Table 1), and the within-subject factor RF type (Non-biological agent, Biological agent) revealed a significant interaction (F_1,20_ = 4.91, *p*<0.05, η^2^ = 0.19). A marginally significant Newman Keuls post hoc test indicated that in the LT group the “Near” space threshold was wider when adopting as RF the biological agent (M = 12.48 m, SD = 6.02 m) compared to the non-biological one (M = 11.65 m, SD = 5.35 m; *p* = .057), replicating previous findings [6]. In the HT group, however, the “Near” space threshold did not differ (*p* = .27) between the adoption of the non-biological agent (M = 11.32 m, SD = 6.33 m) compared to the biological agent as RF (M = 10.86 m, SD = 5.37 m), even if numerically it showed the predicted reversed effect (Fig. 3).

## Discussion

The aim of the current study was to investigate whether being transported in a story with a non-biological agent as a character, leads to a difference on the “Near space” threshold when comparing a non-biological and a biological agent as a reference frame.

In line with our hypothesis the results indicate that participants who transported themselves into the Pinocchio story showed no reliable difference in the Near space threshold when comparing a wooden dummy and an avatar as a RF.

The visually specified environment offers indefinite possibilities for action [17], but we only select the environmental affordances relevant for our action possibilities and intentions to act. The selected affordance drives the scaling of the environment: when I see a cup that affords grasping, I will become a grasper and my arms will be the relevant body part in filtering the space perception, conversely when I see a trail that affords walking, I will become a walker and my legs will be the anatomical part recruited in the space perception [4]. Crucially, an object affords an action only when it is located in our own reaching/peripersonal space [18] or in the peripersonal space of another observed biological agent [19, 20]. At a perceptual level, observing a biological agent able to reach a target with a tool induces to represent the target distance as reduced [21]. The same distance compression is observed when the other agent reaches a very far target with a remote tool (a laser pointer) [21], and even when it is a mere presence within the environment [22]. All these evidences document affordances for another agent, linked to the relevant body part in the specific context, this could be the legs when the agent is within the extended extrapersonal space without tools. As we have indeed shown, a “Near space extension” occurs when participants adopt a biological agent (i.e., an avatar) with available movement potentialities compared to a biological agent unable to walk and to a non-biological agent (i.e., a wooden dummy) as an allocentric RF [6]. We speculated that the observed “Near space extension” is due to the activation of the mental representation of the walking action, which might be implicitly triggered by seeing a biological agent able and prone to cover a distance.

In the present work, we wanted to test whether this subjective Near space categorization can be modulated by inducing the idea that the wooden dummy is a biological agent. Therefore, we investigated the degree to which participants transported themselves into the Pinocchio story. This manipulation was based on previous observations indicating that beliefs about the biological nature of an agent can affect motor co-representation [12, 16]. Crucially, the Near space threshold in the following task was a function of the degree of transportation into the Pinocchio story. As shown by the correlational analysis, the more participants transported themselves into the story, the smaller the difference in the Near space extension when adopting the non-biological vs the biological agent as RF. As a group, participants with the lower transportation scores showed a wider (marginally significant) Near space extension in the avatar condition compared to the wooden dummy, as is typically observed when a biological figure is adopted as an allocentric RF [6]. For participants who transported themselves more in the Pinocchio story (HT group), this effect disappeared. This suggests that when participants identified more with a wooden agent in a story (Pinocchio), the difference between the avatar and the wooden dummy seems to disappear.

However, we don’t know exactly the mechanisms that drive the effect found. By observing the numerical tendency in the two groups (LT, HT) we can speculate that due to anthropomorphizing of the non-biological agent, the biological agent should be processed as “less biological”, with a consequent suppression of her motor co-representation.

Anthropomorphism represents a process of inductive inference about non-human agents by which knowledge about human agents is transferred to non-human agents [23]. Anthropomorphism can be triggered by bottom-up cues, for example similarity to human agents, and/or by top-down information. It responds to our need to make sense of an agent's actions in space, to reduce the uncertainty associated with an agent and to increase confidence in predictions of this agent in the future [23]. Research has shown that anthropomorphism has a profound effect on our social behaviour, and increases interpersonal closeness towards the anthropomorphised object [24].

Another possible explanation is that by inducing the idea of Pinocchio, participants form a strong impression of the non-biological agent, which is then applied to the avatar figure. As such, they see themselves as less similar to the biological agent and consequently co-representations of biological actions could be suppressed. Thus only actions of similar co-actors are simulated [16]. The last speculation is in line with the literature on social categories. Different studies have indeed demonstrated that people categorize the others on the basis of social categories (e.g., gender, race, age) to simplify social information and save cognitive resources [25]. If multiple categories can be applied to the same person, the most dominant one is activated, while the others are suppressed [26]. A very active representation of Pinocchio may temporarily suppress representations of biological agents.

These interpretations, however, are speculative and further research is necessary to support them.

In the future, it should be investigated whether the “Near space extension” also occurs with non-human agents that have equivalent or even higher movement potentialities, or if it is observed only when adopting a human-like agent as RF.

In sum, our research shows that also the extrapersonal space seems to be filtered by the co-representation of potential actions attributed to a RF, that although with a biological appearance, after the manipulation of beliefs, seems to be implicitly perceived as less “living”.
